# CNN-Based Hand Grasping Prediction and Control via Postural Synergy Basis Extraction

**DOI:** 10.3390/s22030831

**Published:** 2022-01-22

**Authors:** Quan Liu, Mengnan Li, Chaoyue Yin, Guoming Qian, Wei Meng, Qingsong Ai, Jiwei Hu

**Affiliations:** 1 School of Information Engineering, Wuhan University of Technology, Wuhan 430070, China; quanliu@whut.edu.cn (Q.L.); mengnanli@whut.edu.cn (M.L.); cyyim@whut.edu.cn (C.Y.); qian_whut@whut.edu.cn (G.Q.); weimeng@whut.edu.cn (W.M.); qingsongai@whut.edu.cn (Q.A.); 2 Wuhan University of Technology Chongqing Research Institute, Chongqing 401135, China

**Keywords:** postural synergy basis, grasping prediction, robot control, convolutional neural network

## Abstract

The prediction of hand grasping and control of a robotic manipulator for hand activity training is of great significance to assist stroke patients to recover their biomechanical functions. However, the human hand and the figure joints have multiple degrees of freedom; therefore, it is complex to process and analyze all the collected data in hand modeling. To simplify the description of grasping activities, it is necessary to extract and decompose the principal components of hand actions. In this paper, the relationships among hand grasping actions are explored by extracting the postural synergy basis of hand motions, aiming to simplify hand grasping actions and reduce the data dimensions for robot control. A convolutional neural network (CNN)-based hand activity prediction method is proposed, which utilizes motion data to estimate hand grasping actions. The prediction results were then used to control a stimulated robotic model according to the extracted postural synergy basis. The prediction accuracy of the proposed method for the selected hand motions could reach up to 94% and the robotic model could be operated naturally based on patient’s movement intention, so as to complete grasping tasks and achieve active rehabilitation.

## 1. Introduction

The hand is one of the most important parts of the human body and it has the characteristics of multiple degrees of freedom and flexible motions. It is difficult to recover the hand functions when the biomechanical and neurophysiological abilities are injured or damaged due to diseases such as stroke. At present, the aging problem in China is serious and the number of elderly aged 60 and over will exceed 300 million by 2025 [1]. The incidence rate of stroke has also increased continuously in the past 30 years, with an average annual growth of 8.3% [2]. Most of these patients have hemiplegic sequelae and may lose part of their physical motion abilities [3]. Disabled hands also experience loss of tactile perception and proprioceptive functions [4]. For patients with hand motion disorders, a robotic manipulator can be controlled with the patients’ own intentions to help them complete some daily tasks, such as grasping motions, which brings great benefits to their recovery and daily lives. In order to naturally control the robot for personalized training, the patient’s grasping activities must be predicted accurately and the robot must be controlled in real-time with a short time delay.

In recent years, new techniques for identifying hand motions have been developed [5,6,7], the application scenarios of which can be divided into two main categories, computer vision and human–robot interactions. Fermuller et al. [8] developed a motion prediction method based on a recurrent neural network which uses the image block around the hand as input and uses the same form to predict the force on the fingertip. Widmann et al. [9] proposed a predictor for online estimation of the target and the time scale of dynamic motion elements using an extended Kalman filter. Shahtalebi et al. [10] proposed a framework to understand the behavior of tremor on various synthetic training data and then identify and eliminate tremor in real time. Hand gesture prediction can predict the instant human activity intention [11] and is a convenient means for the communication between human and machines [12]. The results of hand recognition can be applied to artificial limb control, wearable exoskeleton and human–robot interactions [13]. However, vision-based methods usually have occlusion and delay problems caused by the complexity of hand motions and the limitations of vision-based methods. The kinematic complexity of the human hand makes it challenging to accurately collect and model the information of the human hand, which leads to difficulties in terms of robot control. Commonly used glove-based sensing tends to hinder the user’s interaction and usually requires a long calibration and setup process [14].

Hand gesture recognition methods based on EMG and neural networks, data gloves embedded with sensors and color marking are here compared in depth. Kang et al. proposed a method based on feature calibration to improve the accuracy of gesture recognition based on electromyography (EMG), which is robust to noise [15]. Sheng et al. proposed a single-channel electrode gesture recognition method based on surface electromyography and established an artificial neural network model to mark the observation of sub-windows. The recognition accuracy of this method was 91.3% [16]. Devineau et al. proposed a parallel convolutional neural network for gesture recognition by using the position sequence of hand skeleton joints. The classification accuracy of this model for 14 gesture categories was 91.28% [17]. Lee et al. proposed a real-time dynamic finger gesture recognition method using soft sensors embedded in data gloves. A total of 10 joint angles was measured and each gesture sample took an average of 3 milliseconds [18]. The results show that gesture recognition based on data has higher real-time performance.

In fact, though the movement of the human hand is the result of many joints working together, we tend to use the first action as the principal gesture feature and complete the task via the cooperation of other finger joints [19,20]. Previous research is mainly based on computer vision to use an automatic encoder for neural network training. Encoding and decoding the data can be used to reduce the dimension of the data, but it is necessary to design a complex network structure when training the two sub networks [21,22]. Redundancy still exists in the control of actual manipulators. Qiao et al. proposed a new gesture recognition method based on a principal component analysis (PCA) and the average recognition rate of this method was 93.95% for 9 gestures [23]. It was found that the first two main components of a principal component analysis can reproduce more than 80% of gestures. Santello et al. let the subjects pretend to grasp 57 times and most of the hand motions could be reconstructed from the first two principal components [24]. With the relationship between hand fingers and joints, we can use a group of low-dimensional gesture coordination bases to represent the specific actions of the high-dimensional hand joint space, so as to reduce the dimensions of hand grasping actions and to simplify the control of hand robots. The matrix including principal components extracted from human hand motion data can be called “postural synergy basis”. In practice, when different individuals face the same task, they use similar actions to complete it and the adopted motion mode is highly repetitive, which provides a basis for the rationality of using the postural synergy basis to describe hand motions. In this way, the amount of control inputs for hand grasping activities is greatly reduced and the robotic manipulator can be controlled more naturally with fewer variables.

Using postural synergy basis can decompose and simplify grasping actions, but how to understand the neurologic gesture coordination and how to accurately predict hand actions is still an open problem. The convolutional neural network (CNN) is one of the representative algorithms of deep learning, which is constructed by imitating biological visual perception and imitating the connection between neurons. Hand grasping activities are affected by many factors, such as the shape and weight of the object, or the smoothness of the contact surface. A CNN can effectively predict human hand movements and improve the efficiency of online prediction thanks to its capacity to process complex and high-dimensional data. Shen et al. [25] designed an sEMG-based gesture classification model using a CNN to avoid omission of important features and to improve the recognition accuracy. Yang et al. [26] proposed a CNN-based framework to identify grasping types for understanding the static scene and the fineness of human movements. Edwin et al. [27] presented an approach for dynamic hand gesture recognition to extract features from RGB-Depth data using a CNN. In practice, the recognition of hand grasping actions is complicated; though CNNs provide a solution to classify hand grasping actions, they must be adjusted according to the different patients’ features.

In this paper, a motion intention prediction method based on a convolutional neural network is proposed. The collected motion data of hand grasping activities were input into neural network for gesture prediction. Then, according to the prediction results, the attitude coordination base was extracted from the motion data. By coupling the relationship between finger joints, the reduced-dimension robot model was controlled to ensure the real-time and natural interaction of robot-assisted hand rehabilitation. The main contributions of this paper include the following: (1) By analyzing various hand grasping actions, common hand grasping activities are classified. Three common grasping gestures are selected as the input of the CNN predictor for gesture recognition. (2) An improved CNN-based method for hand grasping activity prediction is proposed to identify hand motions to enable the patient-dominated control of a robotic manipulator for active rehabilitation. (3) An extraction method of attitude cooperation base is proposed which significantly decomposes grasping motion data and reduces the dimension of controlling grasping. A simulated robot manipulator for grasping activity planning and training was designed and posture coordination was used to enhance the real-time and natural control of patients’ active rehabilitation. To the authors’ knowledge, existing studies have not yet reported such a practical solution for various patient-in-charge hand gasping activity training via CNN-based prediction and control via postural synergy extraction. The recognition results in this paper could be used to control various gestures of a modular soft exoskeleton for the active hand rehabilitation of stroke patients [28] and could also be used to control soft rehabilitation gloves to complete daily grasping and drinking movements [29].

The rest of this paper is organized as follows: Section 2 classifies hand grabbing actions and different gestures; the CNN-based hand grasping prediction method is demonstrated in Section 3; in Section 4, we extract the basis of posture coordination to complete hand movement reconstruction. Then, we establish the hand robotic model and realize patient-dominant control for active rehabilitation via postural synergy basis. The results and discussion are presented in Section 5, with the conclusions being drawn in Section 6.

## 2. Hand Grasping Activities’ Classification

When conducting different grasping tasks, the human hand takes different grasping postures and forces from the palm and the fingers. Hand grasping tasks can be roughly divided into two types, force grasping and fine grasping. A standard human hand grasping classification table was established to mine the grasping posture types of the human hand for different objects. Feix et al. proposed a classification method for 33 grasping postures, which combined previous research and different definitions of hand grasping postures. They further refined the grasping classification table and defined the “Grasp” taxonomy according to the sponsored project [30]. The “Grasp” taxonomy is a comprehensive and widely used method in robotics, medicine and biomechanics related to the human hand.

The “Grasp” taxonomy is established on the following four bases: (1) according to the grasping force, as grasping actions can be divided into force type, fine type and the type between the first two; (2) according to the relative position between the object and the human hand, as the hand has pad operation, palm operation and side opposition; (3) according to the number of virtual fingers when fingers work together as a functional unit; (4) the thumb can be abducted or adducted according to the state of thumb.

For patients following neural diseases such as stoke, their hand’s strength and ranges of motion are very limited. To meet the needs of patients, we simplified grasping activities into two types. (1) According to the strength of hand grasping, we divide hand activities into force type, fine type and the type between the first two. The force-type grasping action needs to be carried by the hand with larger grasping force, which means that the contact area between the hand and the object should be larger, so that the object can be grasped more stably. The fine-type action usually does not need a larger grip force, but it needs to accurately complete the motion to grasp the object, which means that the palm may not make contact with the object and the contact area between the hand and the object is smaller. (2) According to the position of the thumb, we define the movements with one being the abduction of the thumb and the other the adduction and these are also related to the position of the palm; when the thumb and the palm are on the same side, the gesture of the hand is thumb adduction and when the thumb is on the opposite side of the palm, the gesture is thumb abduction. Table 1 demonstrates our classification types of hand grasping activities based on the postures.

This study focuses on the situation whereby the patient cannot complete all the actions listed in the classification of hand grasping movements in the process of rehabilitation. In addition, we considered that the hand manipulator cannot complete some too-fine movements. In Section 3, we select several of the most common grasping postures for CNN classification training; further, we select large-diameter action, power-disk action and power-sphere action as the first group; small diameter action, lateral action and palmar action as the second group; and large-diameter action, extension-type action and parallel-extension action as the third group. According to the classification accuracy of the CNN, the influence of grasping posture differences on hand motion classification based on the CNN is explored.

**Figure 1:**
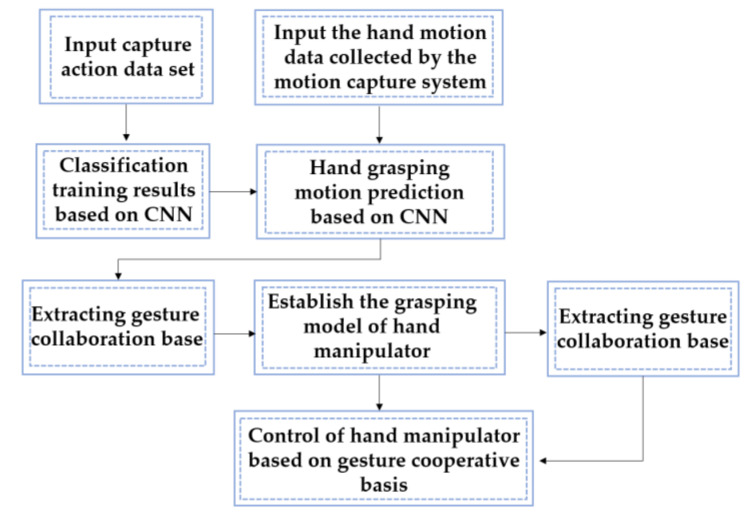
The general architecture of the algorithm and control system.

In order to meet the needs of rehabilitation training for patients with hand movement disorders, we designed a hand manipulator based on hand movement prediction to complete the auxiliary grasping task, as shown in Figure 1. On the basis of gesture classification, large-diameter action, power-disk action and power-sphere action were selected as experimental objects. The motion capture system collected the patient’s hand motion data and input them into the CNN model trained by the online dataset. The motion intention prediction of patients based on the CNN was realized. According to the CNN prediction results, the posture cooperation basis was extracted from the patient motion data. The low-dimensional data of posture cooperation basis were input into the grasping model and grasping planning of the hand manipulator to realize the grasping control of the hand manipulator. Finally, the hand manipulator was controlled to complete a variety of grasping tasks.

## 3. CNN-Based Hand Grasping Prediction

**Figure 2:**
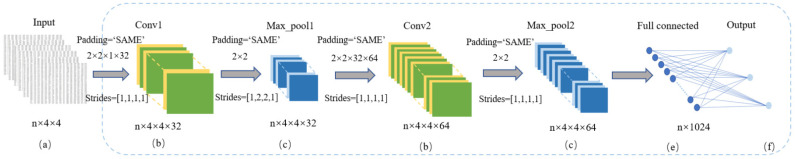
CNN predictor model based on hand motion data.

To predict complex and high-dimensional hand grasping activities accurately, an improved CNN-based classification method is proposed in which higher-level features are used to describe the data to possess better discriminant characteristics. The hand grasping dataset was employed to train and optimize the CNN predictor. Since the data dimension here was not as high as the image, a 2 × 2 convolution kernel was used. We designed two convolution layers, two pooling layers and a full connection layer to classify the three gestures and output the classification accuracy. In addition, the number of iterations was adjusted to avoid the problem of overfitting. The CNN model parameters are shown in Figure 2.

Open datasets of hand grasping motions are available on line. The hand grasping datasets provided by [31] were used, in which the 30 participants were asked to complete grasping tasks using their right hand according to the standard gestures’ classification shown in Table 1. In 33 types of gesture tasks, the subjects were asked to grasp three objects of different sizes or shapes and each test was repeated three times for each object to reduce random errors. The acquisition device used was CyberGlove, which had 16 sensors and collected hand data every 0.02 s interval. A gesture data sample completed in the dataset is shown in Table 2, where the unit of angle of each joint is radian (rad).

Table 2 shows the joint angle data of subjects when completing precision gestures such as pinching paper. When completing the gesture, the CMC, MCP and IP joints of the thumb (T) gradually increased and the ABD abduction joint gradually decreased. The MCP and DIP joints of index finger (I) gradually increased and the joint angles of the other fingers only changed slightly. The data of each gesture were formed into a n × 16 joint angle matrix Q, which was used for data dimensionality reduction and CNN prediction.

*(a) Input Layer*

After the first element time node was deleted for each row of data in the dataset, the rest were 1 × 16 arrays, which represent the angles of hand joints at each moment. In order to facilitate data convolution, we converted the 1 × 16 vector into a 4 × 4 matrix. All the motion data of all subjects in the same grasping posture were extracted from the dataset. As the completion time of each group of gestures was different, we used n to represent the number of sampling points. The n × 16 joint angle matrix Q of each gesture was converted into an n × 4 × 4 matrix as the input of the CNN predictor.

*(b) Convolution Layer*

The function of the convolution layer in a CNN is to extract the corresponding features after calculating the convolution kernel and input data. The convolution layer adopts the way of local connection, so the neurons are connected with the local area in the feature map. The expression of convolution is

(1) $$y^{j}=f\left(b^{j}+\underset{i}{\sum }w^{ij}*x^{i}\right)$$

where $x^{i}$ is the *i*-th input, $y^{j}$ is the *j*-th output, $w^{ij}$ is the shared convolution kernel weight, $b^{j}$ is the feature map offset of the *j*-th output, *f* is the excitation function and ∗ stands for the convolution operation. For the input data with 16 dimensions, a 2 × 2 convolution kernel and two convolution layers were adopted here. The first layer output n × 4 × 4 × 32 feature maps, while the second layer output n × 4 × 4 × 64 feature maps.

*(c) Pooling Layer*

Max pooling selects the maximum value in the neighborhood window to replace the whole values of that window, which can further improve the nonlinear fitting ability of a CNN. It is the most widely used pooling method in CNNs. A 2 × 2 maximum pooling layer and two pooling layers were adopted. The first layer output n × 4 × 4 × 32 feature maps with a step size of 2 × 2 and the second layer output n × 4 × 4 × 64 feature maps with a step size of 1 × 1.

*(d) Excitation Layer*

The role of the excitation layer is to map the data processed by the convolution layer in a non-linear way, which gives the CNN the function of non-linear change and enables the CNN to classify non-linear separable data. The most commonly used function is the ReLU function, which converges faster and the gradient solution is simpler. We chose the ReLU function as the excitation function, whose expression is

(2) $$ReLU\left(x\right)=\left\{\begin{array}{c} x,\;\;x>0\\ 0,\;\;x\leq 0 \end{array}\right.$$

*(e) Fully Connected Layer*

Each node of the fully connection layer is connected with all the nodes of the previous layer and all the features extracted can be integrated. The fully connection layer maps the characteristics of the data to the labels of the samples by weighted summation and then uses the classifier to classify the data. We set the full connection layer with a large number of neurons and then added a dropout layer to reduce the overfitting of the model. We set the number of nodes in the full connection layer to 1024.

*(f) Output Layer*

The output layer is the last layer of a CNN. The *softmax* function is used to control the output ranges from 0 to 1, which denotes the probability of each category. In gesture prediction, the *softmax* activation function is used to generate the probability distributions over three categories. $z_{k}$ is the maximum value of the input and the formula is

(3) $$f\left(z_{j}\right)=\frac{e^{z_{j}-z_{k}}}{\sum_{i=1}^{n}e^{z_{i-z_{k}}}}$$

where *k* is the label of the sample. If multiple data have the same label of the input, making the number of data n, then the loss function of the *softmax* function is

(4) $$loss=-\log\overset{n}{\underset{i=0}{\sum }}e^{z_{k}}-z_{j}$$

Let the output of the *softmax* function be *a*. By deriving the above equation, we obtain

(5) $$\frac{\partial a_{i}}{\partial z_{k}}=\left\{\begin{array}{cc} -a_{i}*a_{k}, & i\neq k\\ a_{k}-a_{k}*a_{k}, & i=k \end{array}\right.$$

To make the output predicted value approach the real value, the model weight was iteratively adjusted. The smaller the cross-entropy loss, the better the model is. So,

(6) $$\frac{\partial loss}{\partial z}=\frac{\partial loss}{\partial a}\frac{\partial a}{\partial z_{k}}=-\left(\frac{\partial loss}{\partial a}\cdot a\right)*a_{k}+\frac{\partial loss}{a_{k}}*a_{k}$$

**Figure 3:**
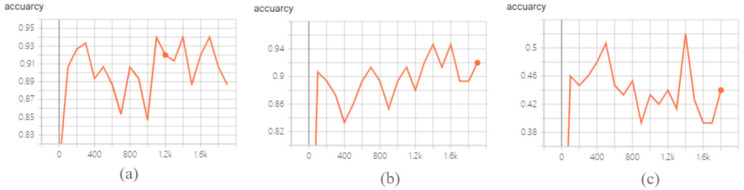
Accuracy changes during CNN prediction training. (**a**) Group 1; (**b**) Group 2; (**c**) Group 3.

This study focuses on the situation whereby the patient cannot complete all the actions listed in the classification of hand grasping movements in the process of rehabilitation. In addition, we considered that the underactuated manipulator cannot complete some too-fine actions. We selected three groups of common hand movements for CNN prediction. The CNN output was transformed into probability distribution by *softmax* to obtain the classification accuracy. Finally, we chose the gesture with the highest probability as our prediction target. In total, 2000 iterative training sessions were conducted for each classification and the accuracy of classification was output every 100 training sessions, as shown in Figure 3a–c.

For Group 1 of hand activities with large-diameter, power-disk and power-sphere actions, the prediction accuracy reached 94%. Small diameter, legal and palmar actions were selected for Group 2, with an accuracy of 89%. The accuracy was only 43% for Group 3, which included large-diameter, extension and parallel actions. This indicates that the CNN-based prediction method was greatly influenced by the differences in grasping postures. According to the prediction results and considering the common hand grasping activities that patients can complete in their daily life, the three groups in Table 3, including the grasp of large-diameter, power-disk and power-sphere objects, were selected for CNN-based hand prediction and control of robot on a postural synergy basis.

In the next section, we present our use of the gesture results recognized by the CNN to control the simulated manipulator to complete grasping tasks, which were formulated according to the recognition results. The patient motion data were input to the CNN for prediction. The dimension of the data was reduced according to the recognized gesture. Finally, the dimension-reduced joint angle data were input into the simulation manipulator to realize the assistive grasping of the simulation manipulator controlled by the patient.

## 4. Control of Hand Robot Model

The prediction results of patient gesture data obtained by the CNN showed the patient’s motion intention, but there were many redundancies in patients’ hand motion data. When controlling an underactuated manipulator, it is necessary to input high-dimensional features for control. In order to simplify the control of the manipulator, we used a principal component analysis (PCA) to reduce the dimension of the patients’ motion data. Finally, we used the dimension-reduced data to control the manipulator, complete the grasping task of the patient and realize the active rehabilitation of the patient.

### 4.1. Relationship among Hand Joints

**Figure 4:**
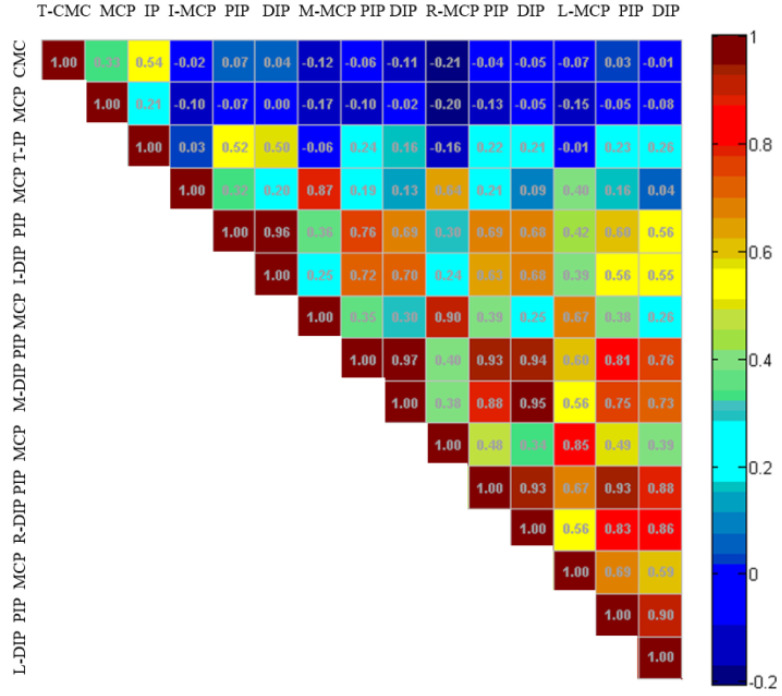
Correlation coefficient diagram of hand joints.

In a grasping task, not all joint angles of the hand are controlled independently of each other. We analyzed the data of all joints in the hand grasping movement dataset, including the joint angle data of 30 subjects repeatedly grasping three objects with different shapes in 33 types of gestures. Finally, the power-disk action in the dataset was selected to draw the correlation coefficients among finger joints, as shown in Figure 4.

The correlation coefficient diagram intuitively shows the correlation among joints. In the power-disk action, the metacarpophalangeal joint (MCP) angles of adjacent fingers were often highly correlated, as were the adjacent proximal interphalangeal joint (PIP) angles. There was a high correlation between the proximal interphalangeal joint (PIP) and interphalangeal joint (DIP) angles of the same finger. The correlation between the thumb joint and the other finger joints was weak. The correlation decreased with the spacing between finger pairs. The positive correlations between joints indicate that the motion of one joint was usually accompanied by the others, considering the coupling characteristics of hand activities. Meanwhile, it was possible to further reduce the dimensions of the hand motion data and accelerate the processing of hand grasping features.

For joints with no obvious correlation, it does not mean there is no coupling relationship; instead, it is possible that a simple linear relationship cannot describe the coupling between these joints. Therefore, more advanced data mining tools are needed, as to obtain the coupling relationship between joints. Since the principal component analysis (PCA) is a common data mining method, which can reduce the actual dimension of data, it was utilized here to extract the principal components with dominant characteristics of hand grasping actions.

The mathematical expression of the PCA is as follows:

(7) $$Q=PC\times W+\bar{Q}$$

where Q represents the real motion data of 15 human joint features and PC denotes the matrix composed of the principal components extracted by the PCA method, with each column representing an extracted principal component. The influence of each principal component on the action is different, so W is defined as the weight coefficient vector; different principal components have different weight coefficients. $\bar{Q}$ is the average value of the sampled action data. The original human hand action can be synthesized by multiplying the weight coefficient by the principal component of the matrix and the average value $\bar{Q}$. Then, (11) can be expanded to

(8) $$\left[\begin{array}{c} q_{1}\\ q_{2}\\ \begin{array}{c} \vdots \\ q_{n} \end{array} \end{array}\right]=w_{1}\times \left[\begin{array}{c} pc_{11}\\ pc_{12}\\ \begin{array}{c} \vdots \\ pc_{1n} \end{array} \end{array}\right]+w_{2}\times \left[\begin{array}{c} pc_{21}\\ pc_{22}\\ \begin{array}{c} \vdots \\ pc_{2n} \end{array} \end{array}\right]+\cdots +w_{m}\times \left[\begin{array}{c} pc_{m1}\\ pc_{m2}\\ \begin{array}{c} \vdots \\ pc_{mn} \end{array} \end{array}\right]+\left[\begin{array}{c} \bar{q_{1}}\\ \bar{q_{2}}\\ \begin{array}{c} \vdots \\ \bar{q_{n}} \end{array} \end{array}\right]$$

where $q_{i}$ represents the angle of the hand joint and the subscript i indicates the *i*-th joint; $pc_{\mathrm{ij}}$ is the joint components of each principal component.

### 4.2. Extraction of Postural Synergy Basis

The postural synergy basis is the dominant principal component with the greatest influence on hand motions. Using the postural synergy basis, we could project the high-dimensional hand data into the low-dimensional space and then complex hand motions from low-dimensional data could be constructed. In order to determine the correlations among the joints, the angle data of 15 joints that followed the time change were paired to calculate the correlation coefficient. $q_{X}$ and $q_{Y}$ represent two arbitrarily selected joints.

(9) $$r=\mathrm{Corr}\left(q_{X},q_{Y}\right)=\frac{Cov\left(q_{X},q_{Y}\right)}{\sigma_{q_{X}}\sigma_{q_{Y}}}=\frac{E\left[\left(q_{X}-Eq_{X}\right)\left(q_{Y}-Eq_{Y}\right)\right]}{\sqrt{E{\left[q_{X}-Eq_{X}\right]}^{2}}\sqrt{E{\left[q_{Y}-Eq_{Y}\right]}^{2}}}$$

The correlation coefficient shows the coupling relationship between two joints. Data dimensionality reduction was achieved by solving the eigenvalues and eigenvectors of the joint angle covariance matrix. For example, we selected the first two principal components to restore the original hand grasping action; then,

(10) $$Q\approx \tilde{Q}=w_{1}\times {\mathrm{PC}}_{1}+w_{2}\times {\mathrm{PC}}_{2}+\bar{Q}$$

**Figure 5:**
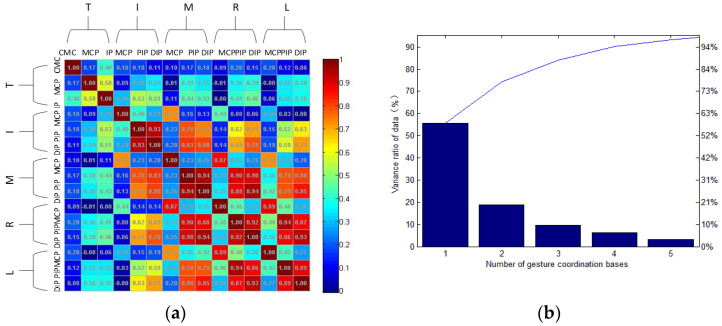
Correlation coefficient diagrams: (**a**) correlation coefficient among joint angles (r); (**b**) change in variance ratio of hand gesture data.

In order to extract the gesture collaboration basis, we calculated the correlation coefficients among the joints in the same gesture grasping task. The correlation coefficient normalizes the covariance matrix to facilitate the observation of the correlation. Figure 5a shows the paired correlation coefficients among 15 joints, forming a real symmetric positive semidefinite matrix. By solving the eigenvalues and eigenvectors of the covariance matrix and selecting the eigenvectors corresponding to the larger eigenvalues, the principal components were extracted.

T, I, M, R and L represent thumb, index finger, middle finger, ring finger and little finger, respectively. The square with color is used to represent the correlation coefficient between two joints, while the colors change from blue to red. Dark blue denotes that the correlation coefficient is 0, while dark red denotes 1. The data between two joints are axisymmetric. The CMC joint, MCP joint and IP joint of the thumb are from left to right and the MCP joint, PIP joints and DIP joints of the other four fingers are from top to bottom. It can be seen that the correlation between PIP joints and DIP joints of four fingers except for the thumb was high (dark red). The correlation between MCP joints of the middle and ring fingers was 0.87 (bright red). The correlation between the PIP joint and DIP joint of the ring finger and the PIP joint and DIP joint of the little finger was also high (bright red), indicating that there were obvious correlations among these joints.

The PCA was used to extract the postural synergy basis. As we tried to retain 95% of the data, the number of extracted principal components and the variance $\sigma_{i}^{2}$ of each principal component are as shown in Figure 5b. The percentage of principal components in the whole information can be calculated by Equation (11).

(11) $$\gamma =\sqrt{\frac{\sum_{i=1}^{k}\sigma_{i}^{2}}{\sum_{i=1}^{n}\sigma_{i}^{2}}}$$

Five principal components were needed to restore the complete hand motion and these five components constituted the postural synergy basis matrix, while the first three principal components postural synergy basis accounted for more than 83% of the sampling data. This shows that, although the hand joint freedom is high for grasping activities, the motion of the human hand can be potentially reconstructed by using the postural synergy basis with fewer data dimensions.

In order to verify the dimension-reduced data, the original gesture could be reconstructed. We used the R-squared coefficient to determine the reconstruction of gesture collaboration basis which is defined as

(12) $$R^{2}=1-\frac{\sum_{i=1}^{n}{\left(q_{i}-\tilde{q_{i}}\right)}^{2}}{\sum_{i=1}^{n}{\left(q_{i}-\bar{q}\right)}^{2}}$$

where $\tilde{q_{i}}$ is the joint angle value reconstructed by postural synergy basis and $q_{i}$ is the joint angle in the original motion; $\bar{q}$ is the mean of the samples. The larger the R-squared coefficient was, the more accurate the reconstructed joint angle.

For different grasping tasks, the first three principal component bases were used to reconstruct the R-squared coefficient of hand motions, as shown in Table 4. Three representative grasping movements were selected as research objects. Among them, due to the obvious linear relationship between PIP joints and DIP joints of four fingers except the thumb, these are not shown in the table. It can be seen from the data in the table that the three selected gesture coordination bases could completely reconstruct the hand movement, which further shows that there was a strong coupling relationship among the joints of the hand in the grasping movement and the grasping task was completed cooperatively.

**Figure 6:**
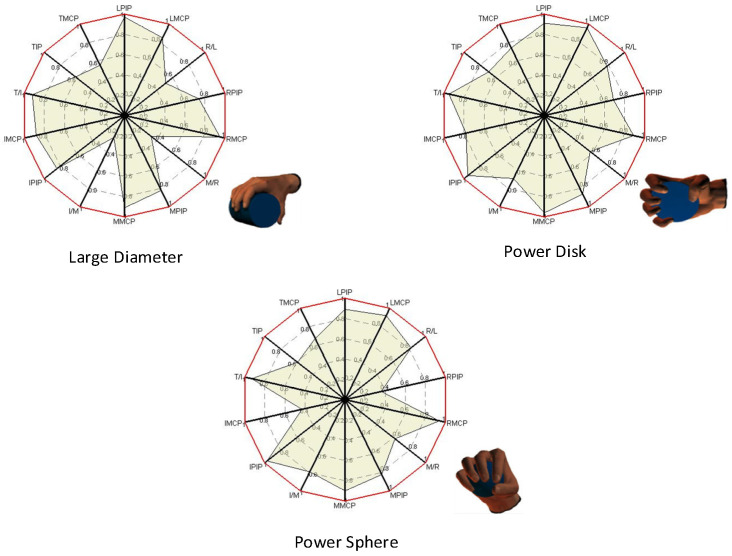
Hand motion reconstruction based on extracted postural synergy basis.

We drew the R-squared coefficient under different actions in Table 4 into a radar diagram as in Figure 6, which can more intuitively show the comparison between the joint angle change of the hand motion reconstructed by the gesture coordination basis and the angle change of each joint in the original motion. Among the three gestures, the reconstruction effect of four fingers was better than that of the thumb. The larger the shadow area of the radar image is, the better the reconstruction effect of all joints of the original gesture was.

### 4.3. Grasping Model of Hand Manipulator

**Figure 7:**
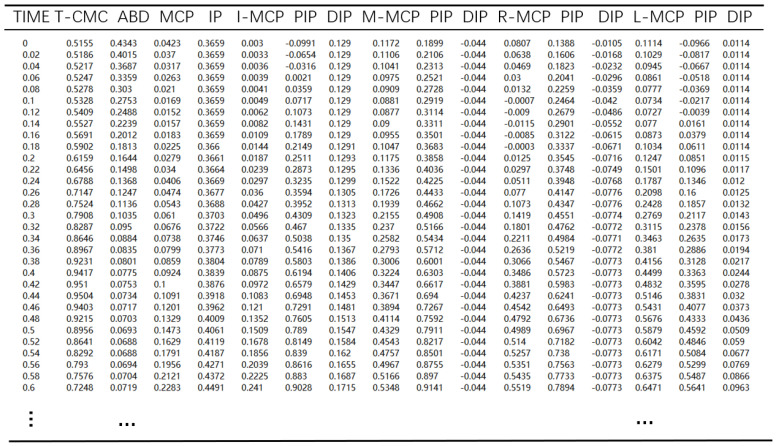
Joint angle data of patient grasping movement.

In order to realize the cooperative task between patient and manipulator, we input the collected patient motion data into the CNN model for gesture prediction. Figure 7 shows the motion data of patients in the large-diameter grasping experiment.

The PCA dimension of patient motion data could be reduced with the output gestures. Low-dimensional joint data could be used well to control the manipulator to assist the patient in grasping the object. In order to achieve real-time control and repeated rehabilitation training, we designed a simulated manipulator to replace the actual manipulator. Patients could repeat training according to pre-designed gesture movements, which made controlling a simulated manipulator more convenient.

**Figure 8:**
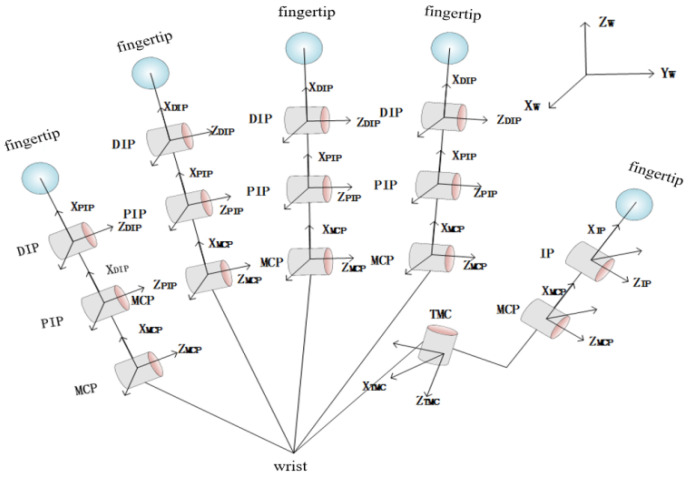
Complete manipulator joint link model.

Based on the extracted postural synergy basis, the grasping models of the under-actuated manipulator could be established. Then, we defined the grasping symbols with the manipulator and object. Each finger of the manipulator could be regarded as a joint link model. The complete manipulator joint connecting rod model is shown in Figure 8.

Let $\left\{N\right\}$ be the inertial coordinate system of manipulator. Let $\left\{B\right\}$ be the coordinate system of the captured object. Let us assume that the number of contact points is $n_{c}$. For the *i*-th contact point, the coordinate system of the local object is $\left\{C_{i}^{o}\right\}$ and the coordinate of the manipulator is $\left\{C_{i}^{h}\right\}$. $\xi_{xy}^{z}$ is the rotational motion of the coordinate system $\left\{Z\right\}$ relative to the coordinate system $\left\{X\right\}$ observed in the coordinate system $\left\{Y\right\}$. $g_{tz}$ represents the attitude of the coordinate system $\left\{Z\right\}$ relative to $\left\{T\right\}$. For $g_{tz}$, we define the adjoint matrix $Ad_{gtz}\in R^{n_{d}*n_{d}}$. $n_{d}$ is the degree of freedom of the system and, in the three-dimensional space system, $n_{d}=6$. The adjoint matrix $Ad_{gtz}\;$ can be transformed as follows:

(13) $$\xi_{xy}^{t}=Ad_{gtz}\xi_{xy}^{z}$$

The symbol $\omega^{z}$ represents the force helix in the coordinate system $\left\{Z\right\}$. Then, in the coordinate system $\left\{T\right\}$,

(14) $$\omega^{t}=Ad_{gtz}\omega^{z}$$

The symbol $\xi_{nb}^{n}\in R^{n_{d}}$ represents the rotational motion of coordinate system $\left\{B\right\}$ relative to coordinate system $\left\{N\right\}$ observed in coordinate system$\;\left\{N\right\}$. We use $u^{n}\in R^{n_{d}}$ to represent the pose of the object in coordinate system $\left\{N\right\}$; then,

(15) $${\dot{u}}^{n}=\xi_{nb}^{n}$$

Similarly, ${\tilde{c}}_{i}^{o}\in R^{n_{d}}$ and ${\tilde{c}}_{i}^{h}\in R^{n_{d}}$ are used to represent the pose of $\left\{C_{i}^{o}\right\}$ and $\left\{C_{i}^{h}\right\}$ relative to coordinate system $\left\{N\right\}$; then,

(16) $$\dot{{\tilde{c}}_{i}^{o}}=\xi_{aC_{i}^{o}}^{C_{i}^{o}}\;\;\;\;\;\;\;\;\;\;\;\;\;\;\;\dot{{\tilde{c}}_{i}^{h}}=\xi_{aC_{i}^{h}}^{C_{i}^{h}}$$

Let us suppose that the manipulator has $n_{q}$ joints; let $q\in {\mathbb{R}}^{n_{q}}$ denote the angle of each joint and $\tau \in {\mathbb{R}}^{n_{q}}$ be the driving torque. Here, we define two concepts, the complete grasping matrix $\tilde{G}$ and the complete manipulator Jacobian matrix $\tilde{J}$. $\tilde{G}\in {\mathbb{R}}^{6_{nc}\times n_{d}}$ maps the motion screw ${\dot{u}}^{n}$ of the object relative to the $n_{c}$ th contact points in the coordinate system {N} and the motion screw $\dot{{\tilde{c}}^{o}}$ of the contact point relative to $\left\{N\right\}$ observed in the local coordinate system of the object is expressed as

(17) $$\dot{{\tilde{c}}^{o}}={\tilde{G}}^{T}{\dot{u}}^{n}$$

$\tilde{J}\in {\mathbb{R}}^{6_{nc}\times n_{q}}$ maps the speed $\dot{q}$ of each manipulator joint to the motion screw $\dot{{\tilde{c}}^{h}}$ of the contact point relative to the coordinate system $\left\{N\right\}$ for the manipulator, which is expressed as

(18) $$\dot{{\tilde{c}}^{h}}=\tilde{J}\dot{q}$$

When both the object and the manipulator are rigid bodies, the motion constraints at the contact points are determined by the contact model. By selecting matrix H, the components to be restricted can be selected from $\dot{{\tilde{c}}^{o}}$ and $\dot{{\tilde{c}}^{h}}$ to form ${\dot{c}}^{o}$ and ${\dot{c}}^{h}$; so,

(19) $$H\left(\dot{{\tilde{c}}^{h}}-\dot{{\tilde{c}}^{o}}\right)={\dot{c}}^{o}-{\dot{c}}^{h}=0$$

Then, the constraint equation of contact point motion is

(20) $$J\dot{q}-G^{T}{\dot{u}}^{n}=0$$

**Figure 9:**
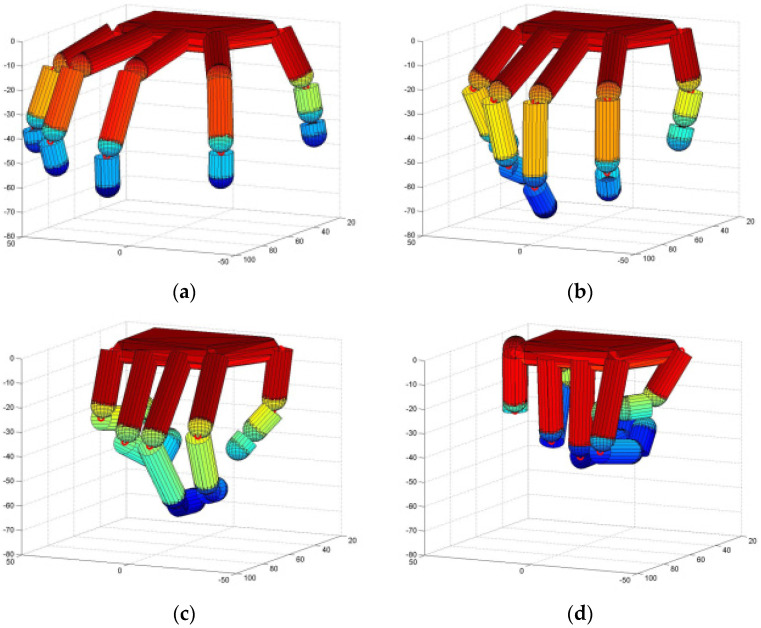
Manipulator grasping based on gesture cooperation. (**a**) Simulation step 1; (**b**) Simulation step 35; (**c**) Simulation step 70; (**d**) Simulation step 100.

The under-actuated manipulator can imitate the human hand to accomplish flexible and diverse grasping actions and its structure design is simple. Based on the simulation toolbox SynGrasp [32] for grasping analysis, we optimized the control of the manipulator according to the CNN output and simulated the grasping task based on the three principal components of pose cooperative selection. The manipulator grasping simulation based on the gesture collaborative basis is shown in Figure 9a–d.

### 4.4. Grasping Action Plan Based on Gesture Cooperative Basis

**Figure 10:**
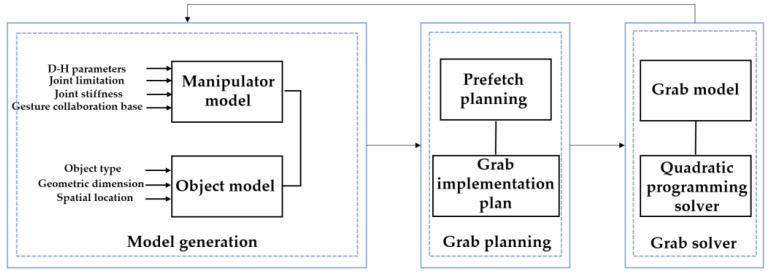
Flow chart of manipulator grasping simulation.

According to the CNN’s prediction results, we selected the grasping activities of large-diameter, power-disk and power-sphere objects as training tasks. We carried out grasping planning and optimization for the manipulator. The flow chart of manipulator grasping simulation and planning based on gesture cooperation is shown in Figure 10.

The simulation system mainly included a model generation part, a grasping planning part and a grasping solver part. Among them, the model generation part mainly constructed the manipulator model by inputting D–H parameters. The object model is determined by its type, size, spatial position and other parameters. The grasping planning part was responsible for determining the pre-grasping stage and grasping implementation stage after obtaining the manipulator model and object model and used the optimal grasping path to realize the grasping task. The grasping solver part was based on the analysis of the manipulator grasping model to solve the model parameters in the grasping process and improve the solution speed of the system.

The complete grasp planning consists of pre-grasping and grasp implementation. In the pre-grasping stage, there is no substantial contact between the manipulator and the object. After obtaining the position of the object relative to the manipulator, the joints move toward the object, but there is no contact point. During grasp implementation, the contact points are calculated to make the grasping stable. In fact, crawl planning can also be regarded as an optimization problem.

(21) $$maxQ=f\left(z,u\right),z\epsilon R^{n_{z}},u\epsilon R^{n_{d}}$$

where $z\epsilon R^{n_{z}}$ maps the actual location of the drive and $n_{z}$ maps the number of gesture collaboration bases. The objective function Q is a complex nonlinear function with multiple constraints. It is difficult to find the solution and it is easy to fall into a poor local optimal solution. However, compared with the fully actuated manipulator, the degree of freedom of the underactuated manipulator based on attitude cooperative basis was significantly reduced, which reduced the dimension of the optimization space, so that it is less difficult to solve the objective function.

We selected a special objective function to evaluate the pre-stage grab quality as

(22) $$Q_{0}=\underset{i}{\sum }\left(\frac{\left|d_{i}\right|}{\alpha }+\left(1-\frac{{\hat{n}}_{i}^{T}\cdot d_{i}}{d_{i}}\right)\right)$$

After the pre-grasping stage, the manipulator starts to grasp and the manipulator further approaches the object until the contact point appears. However, when the contact point first appears, the manipulator cannot grasp the object steadily, so it needs to continue to move towards the object until more contact points appear. This stage is called the first stage of real-time grasping and the optimization objective function is

(23) $$Q_{1}=\sum q_{r,f}$$

where $q_{r,f}$ are the reference angles of the bending joints.

In order to realize the second stage of grasping, we defined a state called force closure, which refers to the force exerted on the object by the manipulator in order to overcome the influence of gravity and other external forces in the grasping process. For example, when we grasp an object to make it stable in the air, this state is force closure. When the grasping force is closed, the first stage of grasping is finished and the second stage starts. The conditions of force closure are as follows:

(24) $$\begin{array}{c} \mathrm{rank}\left(G\right)=n_{d}\\ N\left(G\right)\cap N\left(J^{T}\right)=0\\ \exists \lambda ,\;\omega =-G\lambda \;and\;\lambda \epsilon FC \end{array}$$

It can be obtained, from the static balance equation of the object, that

(25) $$\lambda =-G^{*}\omega +N\left(G\right)\alpha$$

where $G^{*}$ is the generalized inverse of *G* and α is an arbitrary vector. For postural synergy basis-based manipulators, according to formula (20),

(26) $$\lambda ={\left(JS\right)}^{T*}+N\left({\left(JS\right)}^{T}\right)\gamma$$

where $J^{T*}$ is the generalized inverse of $J^{*}$.

(27) $$N\left(G\right)\cap N\left({\left(JS\right)}^{T}\right)=0$$

For the *i*-th contact point, the contact force is $\lambda_{i}$. The volume $\nu =volume\left(GWS\right)$ is used to measure the grasping quality. The shape of the manipulator is further adjusted to improve the grasping stability. The optimization objective function is (32). We summarize the important mathematical symbols and their significance in Nomenclature.

(28) $$\lambda_{i}=\overset{n_{g}}{\underset{j=1}{\sum }}\alpha_{i,j}f_{i,j}$$

(29) $$\alpha_{i,j}\geq 0,\overset{n_{g}}{\underset{j=1}{\sum }}\alpha_{i.j}=1$$

(30) $$\omega_{i,j}=\left[\begin{array}{c} f_{i,j}\\ 1/r\left(d_{i}\times f_{i,j}\right) \end{array}\right]$$

(31) $$GWS=ConvexHull(\overset{n_{c}}{\underset{i=1}{\cup }}\left\{\omega_{i},1,\ldots ,\omega_{i,n_{g}}\right\})$$

(32) $$Q_{2}=volume\left(GWS\right)$$

**Figure 11:**
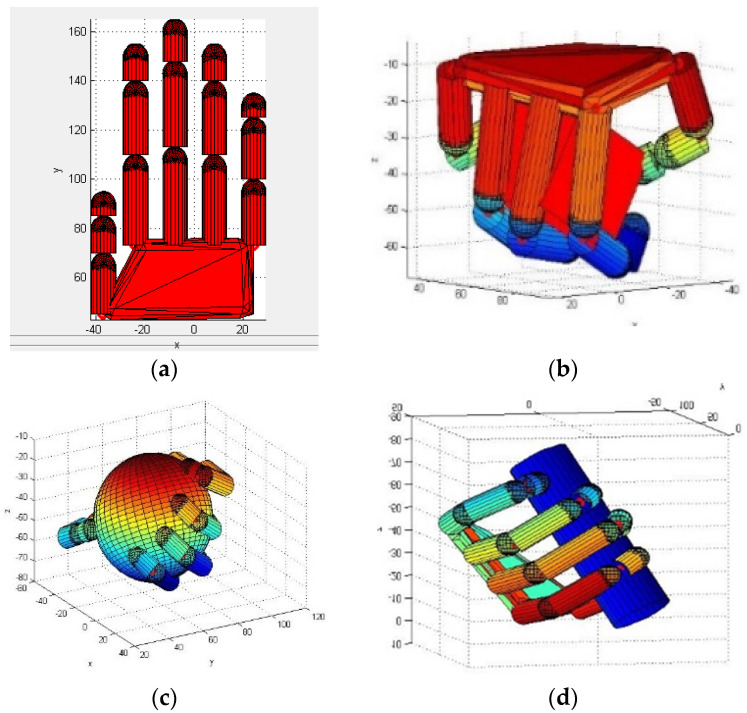
Simulated manipulator and grasping actions for different objects: (**a**) under-actuated hand manipulator, (**b**) grasping of a square object, (**c**) grasping of a spherical object and (**d**) grasping of a rod-shaped object.

We defined a 15-degree of freedom (DOF) under-actuated manipulator in the SynGrasp toolbox and its kinematics model was established by a D-H method, as shown in Figure 11a. The grasping task was decomposed into lower data dimensions and the principal component was selected as the input of manipulator control. Using the simulated manipulator, the grasping planning for three kinds of objects (square object, spherical object and rod-shaped object) was implemented and the manipulator was then controlled to complete the grasping task based on the postural synergy basis, with the results shown in Figure 11b–d.

## 5. Experiments and Results Discussion

**Figure 12:**
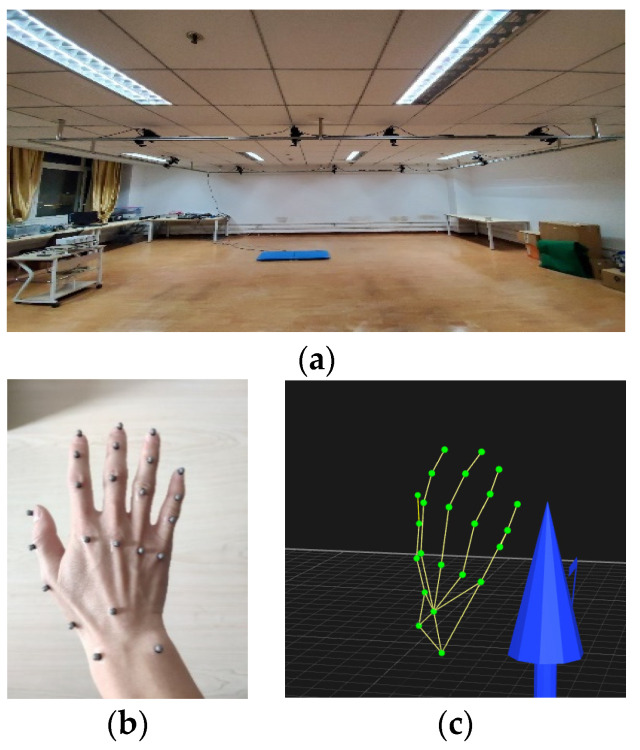
Motion capture system: (**a**) system setup, (**b**) distribution of the markers on hand joints and (**c**) hand models based on the markers.

In order to verify the performance of the CNN network and realize the real-time control of manipulator, hand movement data were collected via the Qualisys motion capture system and the grasping actions were predicted via the CNN predictor. Our system configuration is shown in Figure 12a. A total of eight infrared lenses and one video lens formed the hand motion capture system. Markers with 3 mm diameters were evenly distributed in each joint of the hand, as in Figure 12b. Each finger was attached with four markers. Moreover, one was attached to the back of the hand and two were attached to both sides of the wrist, with the reconstructed hand model in Figure 12c.

The subjects were asked to complete three kinds of actions, large diameter, power disk and power sphere, for three objects, cylinder, disk and ball, each of which was repeated three times. The subjects sat on the chair in a relaxed state and the markers on their right hands were set with the configuration shown in Figure 12. Once the test started, the subjects began to use their right hand to perform a grasping activity. The motion capture system worked at 100 Hz and each test took 10 s, while Qualisys managing software was utilized to record hand data. This trial was approved by the Human Participants Ethics Committee from the Wuhan University of Technology and written informed consent was obtained from each participant.

**Figure 13:**
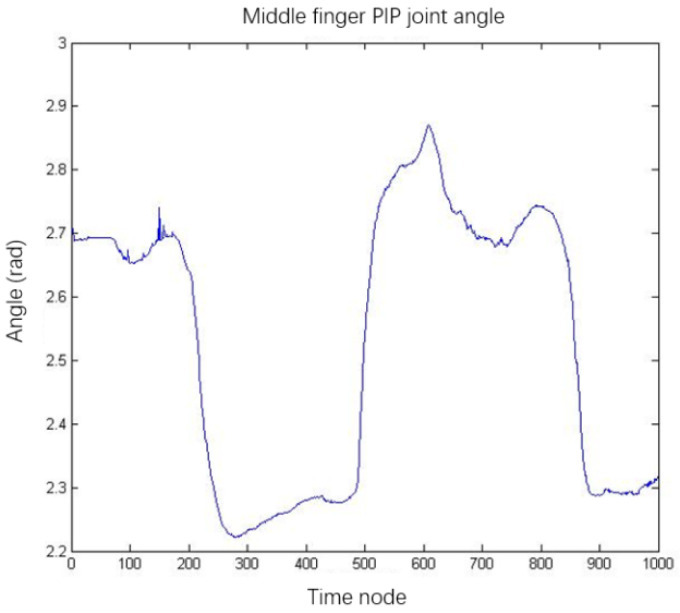
Middle finger PIP joint angle.

**Figure 14:**
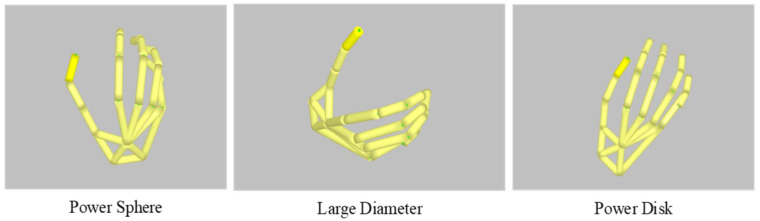
Grasping motions predicted from captured data.

Figure 13 shows the angle change of the middle finger PIP joint when a large-diameter grasping action was completed after filtering. We output the angles of all joints to the same file so that they were arranged in the same format as the dataset used in Section 3. Based on the hand data collected by the motion capture system, the predicted grasping actions using the CNN are shown in Figure 14.

**Figure 15:**
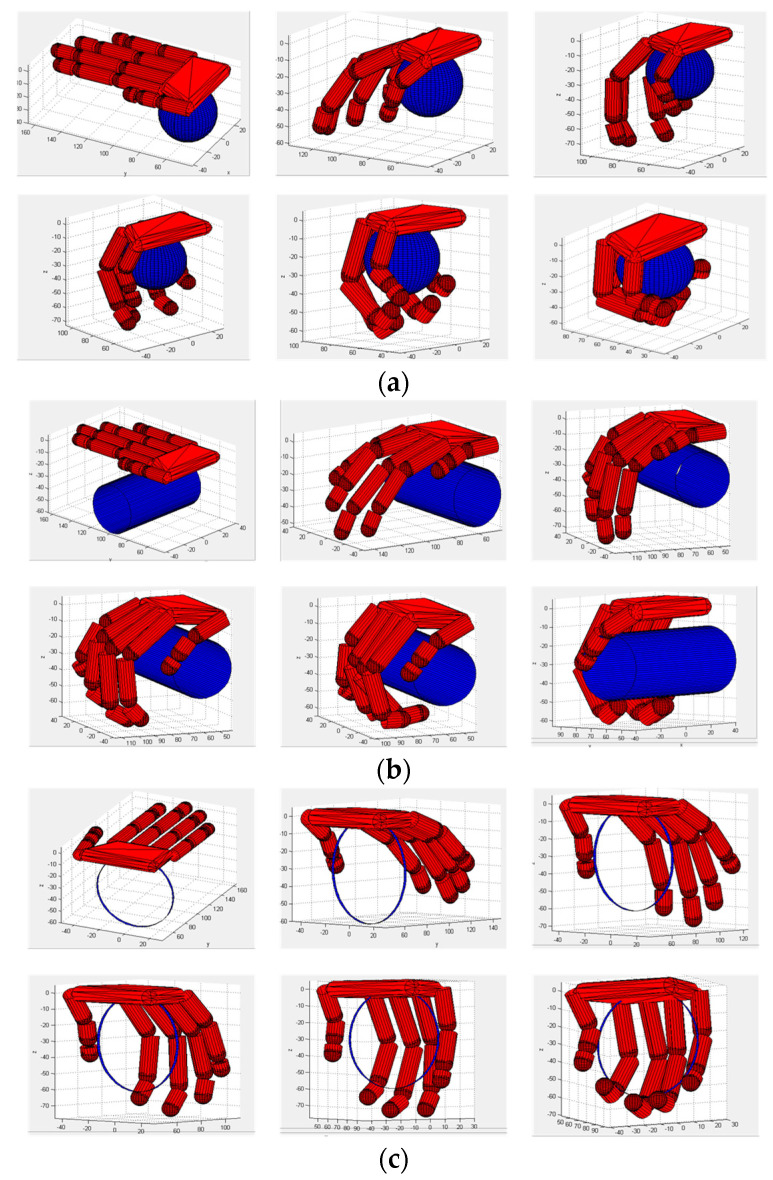
Control of manipulator model to complete grasping tasks: (**a**) power-sphere motion, (**b**) large-diameter motion and (**c**) power-disk motion.

Then, we used the predicted motion results to control a robotic manipulator for patient-dominant active hand training. The simulation model of the under-actuated manipulator was established and the performance of CNN-based grasping activities prediction was verified. As shown in Figure 15a–c, three types of hand motions, large-diameter, power-disk and power-sphere, were accurately recognized by integrating the postural synergy basis and the CNN predictor. Then, the virtual robotic manipulator was controlled to complete the tasks and grasp the test objects firmly. This validates that the proposed method based on the CNN and low-dimensional postural synergy basis could accurately predict hand grasping actions and effectively control a robotic hand manipulator model for active hand rehabilitation.

The excessively high complexity of the human hand brings great difficulty to the analysis and prediction of hand movements, which makes the control of hand robots difficult. The use of postural synergy basis not only can decompose and simplify grasping activities, but can also decompose complex hand actions into a lower dimension, so as to control the manipulator to complete grasping tasks with less input. Hand motion prediction is the basis for patient-dominant robot-assisted rehabilitation, which can be used to optimize the rehabilitation experience and avoid secondary injury. Common prediction methods include the use of biological signals and image recognition. In [33], an individual with traumatic high-cervical spinal cord injury coordinated grasping movements with his own paralyzed arm and hand. However, the equipment for biological signals is usually complex and expensive. Vision-based methods have the advantages of convenient interaction and rich expression [34], but most of them are difficult to apply in the real world. For hand motion data, a CNN is appropriate for dealing with high-dimensional and complex data, which can improve the efficiency of online prediction and the real-time performance of the whole system.

Based on the classification of hand grasping activities, a CNN-based prediction method is proposed in this paper. It is worth mentioning that the types of hand grasping movements were adjusted according to the patients, which is more in line with the daily needs of the patients. Taking the under-actuated manipulator as the control object, the postural synergy basis extracted from hand grasping motions was used to reduce the hand data dimensions, so as to simplify the control input of the manipulator. Li et al. [35] used a 3D convolutional neural network to classify gestures which could extract spatio-temporal features and achieve a recognition accuracy of 65.35%. Our method could reach a higher accuracy, up to 94%, even for dynamic hand motions. Huu et al. [36] designed an algorithm to recognize gestures in home applications, which utilized an artificial neural network (ANN) to help users interact with a computer. However, the accuracy rate largely depended on the number of training images (videos) and their resolutions.

For robotic hand control, traditional hand-gesture-based methods are complicated. One of the challenges in robotic grasping is how to coordinate so many finger joints in order to generate an appropriate grasping posture for a specific object [37]. To resolve this problem, the complexity of hand control can be reduced by using the synergy basis. Liu et al. [38] proposed a postural synergy-based method for a multi-joint upper-limb exoskeleton rehabilitation robot. Similarly, a synergy-based strategy for UB Hand IV planning strategy was proposed in [39] to simplify the grasping synthesis and dimensions. However, these studies only work well on specific manipulators. There is little work on under-actuated robot manipulator control for patient-dominant rehabilitation assistance. In this paper, the postural synergy basis of hand motions was extracted to simplify the computations for control and a CNN-based hand activity predictor is proposed to control any robotic models based on the postural synergy basis for hand rehabilitation training.

## 6. Conclusions

By extracting the postural synergy basis of hand motions, this paper explores the cooperative relationship in the hand grasping action, simplifies the hand grasping action and reduces the hand data dimensions. Based on the improved CNN predictor, the hand grasping motion category could be predicted with an accuracy up to 94% for the selected activities. A simulated robotic hand manipulator was established and controlled on the postural synergy basis to help patients complete the grasping tasks according to the prediction, so that patient-in-charge assistance could be realized. The experimental results prove that the proposed prediction algorithm could predict the motion intention of the patients and the control method based on postural synergy could help them complete the grasping action easily. In the future, subsequent research will be conducted on the prediction of more hand movements for daily life activities and intelligent robot-assisted rehabilitation. We will further improve the control structure of the manipulator, so that the robot can better assist patients in patient-in-charge training and daily life. Meanwhile, the recognition results of this paper can be used to control patients to perform interactive tasks in a virtual/enhanced environment and to carry out rehabilitation training by repeatedly grasping objects in the virtual scene. It can also be used to control soft rehabilitation gloves to aid daily grasping behavior and complete drinking actions. The proposed method can also be combined with remote rehabilitation to help patients realize prediction and control of hand movement in residential settings.

## Figures and Tables

**Table 1:** Classification of hand grasping motions of patients.

Type	Power	Intermediate	Precision
Thumb abducted	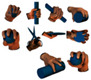	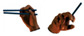	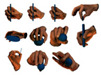
Thumb adducted	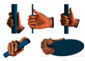	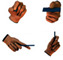	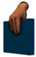

**Table 2:** Joint angle data in open dataset.

TIME	T-CMC	T-ABD	T-MCP	T-IP	I-MCP	I-PIP	I-DIP	M-MCP	⋯
0	0.394	0.3957	0.2966	−0.0101	0.1408	0.0345	0.0489	0.1689	⋮
0.02	0.3998	0.3697	0.2815	−0.0135	0.134	0.0345	0.0514	0.1749	
0.04	0.4056	0.3437	0.2664	−0.0169	0.1272	0.0345	0.0539	0.1809	
0.06	0.4114	0.3177	0.2513	−0.0204	0.1204	0.0345	0.0563	0.1869	
0.08	0.4172	0.2917	0.2361	−0.0238	0.1136	0.0345	0.0588	0.1929	
0.1	0.4288	0.2635	0.2207	−0.0289	0.1048	0.0345	0.0613	0.194	
0.12	0.4463	0.2348	0.1989	−0.0359	0.0933	0.0345	0.0639	0.1886	
0.14	0.4706	0.2061	0.1725	−0.0451	0.0795	0.0345	0.0666	0.1771	
0.16	0.5016	0.1778	0.144	−0.0564	0.0637	0.0346	0.0694	0.1609	
0.18	0.5385	0.1503	0.1157	−0.0694	0.047	0.0347	0.0721	0.1413	
0.20	0.58	0.1244	0.0896	−0.0837	0.0305	0.0352	0.075	0.1201	
⋮	⋯								

**Table 3:** Classification accuracy of three groups of hand grasping motions for CNN-based prediction and control.

Group	Motions			Accuracy
Group 1	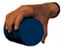	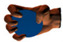	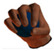	94%
Group 2	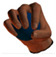	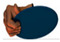	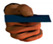	89%
Group 3	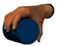	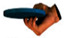	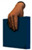	43%

**Table 4:** R-squared coefficient of hand motion reconstructed by three postural synergy bases.

	Motion 1	Motion 2	Motion 3	Average
$T_{mcp}$	0.55	0.71	0.67	0.65
$T_{ip}$	0.61	0.69	0.59	0.63
T/I	0.92	0.95	0.93	0.93
$I_{mcp}$	0.88	0.78	0.43	0.70
$I_{pip}$	0.84	0.95	0.97	0.92
I/M	0.24	0.69	0.79	0.57
$M_{mcp}$	0.91	0.96	0.90	0.92
$M_{pip}$	0.82	0.86	0.82	0.83
M/R	0.33	0.57	0.62	0.51
$R_{mcp}$	0.95	0.89	0.94	0.93
$R_{pip}$	0.74	0.69	0.36	0.60
R/L	0.51	0.78	0.82	0.70
$L_{mcp}$	0.85	0.96	0.92	0.91
$L_{pip}$	0.97	0.91	0.89	0.92

## Nomenclature

$\left\{N\right\}$	coordinate system fixed to workspace
$\left\{B\right\}$	coordinate system fixed with object
$\left\{C_{i}^{o}\right\}$	reference system of an object with the *i*-th contact point as the origin
$\left\{C_{i}^{h}\right\}$	reference system of manipulator with the *i*-th contact point as the origin
$n_{c}$	number of touchpoints
$n_{d}$	grab system dimensions
$n_{l}$	dimensions of all contact motion constraints
$n_{q}$	number of joints of manipulator
$n_{z}$	number of gesture collaboration bases
${\tilde{C}}_{i}^{o}\in R^{n_{d}}$	relative pose of $\left\{C_{i}^{o}\right\}$ and $\left\{N\right\}$
${\tilde{C}}_{i}^{h}\in R^{n_{d}}$	relative pose of $\left\{C_{i}^{h}\right\}$ and $\left\{N\right\}$
$u\in R^{n_{d}}$	relative pose of object and $\left\{N\right\}$
$\;\omega \in R^{n_{d}}$	a spiral of external force acting on an object
$q\in R^{n_{q}}$	actual manipulator joint angle
$q_{r}\in R^{n_{q}}$	reference manipulator joint angle
$\tau \in R^{n_{q}}$	torque of each joint of manipulator
$\lambda \in R^{n_{l}}$	contact force between manipulator and object
$z\in R^{n_{z}}$	actual location of the drive
$\sigma \in R^{n_{z}}$	driving torque
$G\in R^{n_{l}\times n_{d}}$	grab matrix
$J\in R^{n_{l}\times n_{q}}$	Jacobian matrix of manipulator
